# First Report of the Royal Metropolitan Infirmary for Sick Children, Read at a General Meeting of the Governors and Directors of That Charity, Held at Saville House, Leicester-Square, on Wednesday, 24th April, 1822; the Right Rev. the Lord Bishop of Chester in the Chair

**Published:** 1822-08

**Authors:** 


### STATISTICAL MEDICINE.

Art. I.—*First Report of the Royal Metropolitan Infirmary for Sick Children, read at a General Meeting of the Governors and Directors of that Charity, held at Saville House, Leicester-square, on Wednesday, 24th April,* 1822*; the Right Rev. the Lord Bishop of Chester in the Chair.*

In compliance with the request of the committee, your medical officers have the honour to lay before you the First Report of their practice, together with such a general account of the operations of the Institution as may prove interesting to the governors at large. They feel particular pleasure in discharging this duty, because they find, on comparing the original address to the public with the results which have since taken place, that the utility of the charity has exceeded even the high expectations of its founders; and is likely, on many accounts, to prove a source of benefit, not only to the immediate objects of relief, but to the community.

In giving the following concise medical and surgical history of the patients, your medical officers beg to call your attention to the registers kept at the different stations; from the plan and arrangement of which, peculiar to your institution, they are enabled to lay before you, not merely a numerical return of admissions, but also a brief history of the circumstances connected with each patient; such as the place of residence, the part of the house occupied, the number of children in the family, the occupations of the parents, the nature of the complaint, the number of visits made at your Infirmary for the cure, the result of the treatment, and, lastly, the history of each individual with respect to small-pox and vaccination. The manner in which these registers have been kept by the medical clerks, is such as must meet the approbation of the governors; and render them public documents of value, enabling accurate calculations to be made on an extensive scale, concerning the comparative frequency of different diseases, and the influence produced upon them by situation, the trade of the parents, and other questions relating to statistical medicine. One of these appears of so much importance, that we beg permission briefly to point it out. The total number of patients admitted at the different stations amounts to 8475; of these, only 3446 appear to have been vaccinated, 1884 having had the small-pox, and, consequently, 3145 remain totally unprotected; a proportion of children liable to small-pox which cannot be contemplated without much anxiety, and which would probably render the fatality of that disease very great, should it unfortunately become epidemic. In compliance with the objects of the institution, your medical officers have endeavoured to remedy this evil; but they regret to say that few parents avail themselves of the offers to vaccinate their children. Of those who have been affected with small-pox, 1360 have had it in the natural form, and 524 have been wilfully subjected to it by inoculation; giving lamentable evidence that there are still members of the medical profession who employ themselves in creating those maladies which the exertions of their brethren and the legislature are alike directed to suppress.

| | |
|---|---|
| In the medical department, the number of patients admitted amounts to | 6053 |
| Of these have been treated by Dr. Granville, at the Central Station, from January 1821 to 1st April 1822,—a period of fifteen months | 1932 |
| By Dr. Macleod, from 20th Sept. 1820 to 1st April 1822,—eighteen months | 2559 |
| By Dr. Hutchinson, from November 1820 to 4th October 1821 | 711 |
| By Dr. Webster, from October 1821 to 1st April 1822 | 851 |

During the winter of 1820-1821, our attention was particularly attracted by the number of children affected with inflammatory diseases of the chest, which generally run a severe, and in some instances a fatal, course. Although this remark applies to all the stations, yet the severity of these diseases was particularly displayed in lower Westminster; the lowness of the situation, and its proximity to the river, rendering it peculiarly unfavourable to complaints connected with respiration. The present season has been remarkable for its mildness, and a corresponding change may be perceived in the prevailing diseases; inflammatory attacks having been both milder and less frequent, putting on the appearance of simple catarrh. Measles and hooping-cough have been for some months, and continue still, epidemic; but they, in general, assume a favourable character, and have rarely proved fatal. The class of diseases most uniformly prevalent, and apparently least influenced by variations of temperature and the state of atmosphere, are those connected with digestion. These complaints, prevalent in every class of society, are particularly so among the children of the poor, who are generally fed, from the period of their weaning, upon the same meagre and unwholesome diet as their parents, giving rise to mesenteric obstruction, one of the most fatal among the diseases of children.

In the annexed tables we have endeavoured to give such a view of the cases treated at the infirmary as might be intelligible to those unacquainted with our profession. The fatal cases, it will be seen, have amounted to 147; a number of deaths which, to those ignorant of the peculiar economy of your institution, must appear extremely small. The truth is, however, that this account cannot be taken as indicative of the exact mortality. The doors of the infirmary are shut against no applicant; the consequence of which is, that many of those who get well never take the trouble of acknowledging the benefits they have received: still less do those whose children sink under their complaints think themselves called upon to return thanks for treatment which has proved unavailing. To this circumstance is likewise to be attributed the great proportion of cases, the events of which are unknown; but, although an obstacle is thus presented to a more definite history of the results, yet your medical officers feel assured, from the nature of the cases, that the great majority of those who have discontinued their visits, have done so from the recovery of their children; and that not only the apparent, but the real, mortality is much less than they had anticipated, or than would be the case under any other arrangement. In other institutions, much time is lost from the difficulty of procuring a recommendation; and the little patient is generally subjected, in the first instance, to the trial of some domestic, and often deleterious, nostrum; whereas the early detection of disease is often of so much importance, that no future skill can compensate for its loss. The facility, therefore, of obtaining advice at your institution becomes of inestimable value to the poor, and more than counterbalances any inconveniences to which it may give rise.

Much of the above reasoning will equally apply to the surgical department of your excellent institution. It only now remains, therefore, for us to lay before you a table of the prevailing surgical diseases, and to offer such remarks as may arise out of it, and also out of the various columns in the Register, as it may be interesting to know, or as may be more immediately connected with this branch of medical practice.

It will be observed, by a reference to the table B, that a great majority of the children, treated by the surgeons, have laboured under cutaneous diseases and scrofula; and we have noticed these diseases, with ophthalmia, to prevail most, or be most inveterate, where poverty and want of cleanliness were the greatest; and also where the ground, or under-ground, part of the house was that occupied by these individuals. Of the former, porrigo seemed to take the lead, more particularly that species of it termed scald head; and, as this is a contagious disease frequently untractable and difficult of cure, as well as rapid in its dissemination, society at large must have derived considerable benefit from the existence of your institution, by the early check it has opposed to the propagation of this malady among all classes of the community. Of the other cutaneous diseases, itch and the varieties of ring-worm have been the most frequent; but some highly interesting cases of erysipelas of infants have also occurred.

Under the head of Scrofula we have, for the sake of brevity, included diseases of the hip, knee, ankle-joints, and curvatures of the spine, &c. as well as glandular swellings. Cases of great moment, in some of this division, have been treated by the surgeons, and, they are happy to say, with more success than could reasonably be expected, considering that the little patients were less strictly watched or confined to their beds than would have been judged proper within the walls of an hospital. Ophthalmia in all its varieties, abscesses, ulcerations, scalds, burns, and accidents, rank next in frequency; but upon which we have not any remark to make worthy of particular notice.

Surgical operations of importance have been performed, as frequently as the cases requiring them came under the management of the surgeons: particularly a case of stone in the bladder, where the operating surgeon on the occasion had to provide for the patient at his own expence, including lodging, nurse, and provisions, until the boy's recovery; a striking instance of the necessity that exists for the speedy adoption of the hospital part of the plan of this extensive charity, and to which we beg earnestly to call the attention of the meeting and the governors at large.

One of the features peculiar to this institution is the plan, for which we are indebted to our very assiduous treasurer, Mr. Harriss, of using tokens as the representatives of money in the payment for the medicines. Sufficient time has now been afforded to judge of this plan, and your medical officers have much pleasure in bearing testimony to its utility. It appears to them to have all the advantages of a ready-money transaction, preventing the possibility of imposition, while it is not attended with any of those inconveniences which some apprehended from its adoption. By this arrangement, in conjunction with others, the machinery of the institution is much simplified: in proof of which it is enough to mention that, at each station, above an hundred patients are not only seen and prescribed for, but have their medicines made up, paid, and delivered, in the course of the forenoon.

One great desideratum, however, yet remains, without which the benevolent wishes of the governors, and the exertions of the medical officers, must be in a great measure frustrated: we mean an establishment for the reception of those children whose complaints, whether medical or surgical, demand active measures and assiduous care. Those most acquainted with the habits of the poor,—their procrastination in the first instance, and their subsequent abandonment of all remedies from the idea that they must prove unavailing,—know best the difficulties which medical men frequently find in enforcing the application of the necessary means. And, from the want of means, and in many instances of proper accommodation, the parents of poor children cannot provide sustenance, separate beds, or even a separate space in their apartments, for them: neither can they devote the time and attention indispensably necessary to their treatment, when the family is numerous. Nor is this exigency at all provided for by any public institution; the regulations of all the hospitals in the metropolis rendering the admission of children nearly impossible. It is generally acknowledged that the diseases of children are less understood than those of adults; a circumstance in a great measure owing to the latter being brought under the immediate eye of medical practitioners in public hospitals and infirmaries, where an opportunity is afforded of watching the appearances of disease and the effect of remedies. Nor can it be doubted that much of the obscurity attending diseases of the earlier periods of life would be removed, were similar advantages possessed; thus conferring indirectly a benefit on the community, the value of which every parent must appreciate. We would desire, therefore, most earnestly to direct the attention of the governors to this point, set forth at the original foundation of the charity as one of its primary objects. In answer to all objections, we appeal to the experience of other nations, among whom the advantages of such establishments are not speculative, but great and practical blessings to the children of the poor.

So widely have the benefits of your institution, although still in its infancy, been diffused, that there is no parish in the western part of the metropolis which has not furnished some hundreds of patients who have been relieved by it. Scarcely a street in which some children may not be found now enjoying the blessings of health, who, but for the existence of this charity, must necessarily have been abandoned to the miseries of neglected disease. It would be easy for your medical officers to present the governors with highly-coloured pictures of suffering, and to excite their sympathy by the relation of some of those scenes of misery to which they are called in the exercise of their profession; but their appeal is to the judgment, not the feelings. The registers of the institution, although so recently established, bear record of relief afforded to 8475 children, many of whom must otherwise have perished unassisted. This presents an argument in favour of its utility which no panegyric of ours could strengthen, and which affords an unanswerable proof of the discernment of those who first suggested its establishment. Indeed, if we consider the tendency which this institution has to improve the general management of children,—of checking, in their commencement, those acute diseases which are dangerous to life, and arresting the progress of those lingering maladies which sow the seeds of future decrepitude, rendering the patients miserable to themselves and burdensome to the community,—we believe few charities will be found to possess stronger claims upon the support of the humane, or the protection of the government.

*(Signed by all the Medical Officers of the Institution.)*

*24th April*, 1822.

### (A.) TABLE OF MEDICAL CASES.

| *Nature of the Diseases.* | |
|---|---|
| Diseases referable to the Head | 386 |
| ——— Chest | 1239 |
| ——— Mouth, Throat, &c. | 349 |
| ——— Abdomen | 1771 |
| With acute Eruptions | 687 |
| Febrile Diseases, not easily referable to particular organs | 332 |
| Diseases referable to various parts, — viz. Rheumatism, Scrofula, &c. | 1289 |
| Total | 6053 |

| *Results.* | |
|---|---|
| Cured, returned thanks | 2964 |
| Discontinued attending, in the great majority of cases from convalescence | 2210 |
| Have died | 139 |
| Under treatment | 740 |
| Total | 6053 |
| Vaccinated (previously) | 2451 |
| Had Small-pox | 1271 |
| Unprotected by either Small-pox or Cow-pox | 2321 |
| Total | 6053 |

| | | |
|---|---|---|
| At Central Station, in fifteen months, treated by Dr. Granville | | 1932 |
| At Westminster Station, in eighteen months, | by Dr. Macleod, 1564<br>by Dr. Webster, 851 | 2415 |
| At Mary-le-bone Station, in eighteen months, | by Dr. Macleod, 995<br>by Dr. Hutchinson, 711 | 1706 |
| Total medical cases | | 6053 |

### (B.) TABLE OF SURGICAL CASES.

| *Nature of Diseases.* | | *Results.* | |
|---|---|---|---|
| Cutaneous Diseases ........ | 1010 | Cured, including those who have not returned thanks .. | 2036 |
| Scrofula.................... | 458 | Died ........................ | 8 |
| Ophthalmia ................ | 227 | Remain under treatment .... | 378 |
| Erysipelas ................ | 50 | | |
| Abscesses .................. | 171 | Total of results.... | 2422 |
| Ulcerations, Caries, &c. .... | 99 | | |
| Scalds, Burns, Accidents .... | 98 | | |
| Other complaints ............ | 309 | Vaccinated (previously)...... | 995 |
| | | Had Small-pox ............ | 613 |
| Total of admissions.... | 2422 | Unprotected by either....... | 824 |

| | |
|---|---|
| Treated at Central Station, by Mr. A. Copland Hutchison, from Jan. 1821, to April 1822 ........ | 799 |
| Treated at Westminster Station, by Mr. Money, from Sept. 1819, to 1st April, 1821 ........ | 1032 |
| Treated at Mary-le-bone Station, by Mr. Money, from 20th Sept. 1820, to 5th May, 1821 ........ | 104 |
| Treated at ditto, by Mr. R. W. Bampfield, from 5th May, 1821, to 1st April, 1822 ........ | 480 |
| Total........ | 2422 |

TABLE C.—*Number of Patients admitted during the Period embraced in the Report according to Parishes.*

| | | | |
|---|---|---|---|
| St. Andrew's ............... | 45 | St. James's ................ | 506 |
| St. Ann's .................. | 842 | St. Giles's................. | 665 |
| Adelphi.................... | 1 | Knightsbridge .............. | 1 |
| Aldgate ................... | 1 | Lambeth .................... | 39 |
| Blackfriars................ | 2 | St. Luke's.................. | 6 |
| Bayswater ................. | 8 | Kilburn .................... | 1 |
| Christchurch .............. | 1 | Lisson Grove ............... | 6 |
| Clapham ................... | 1 | Mary-le-bone ............... | 2117 |
| Chelsea ................... | 8 | St. Margaret................ | 1357 |
| Clerkenwell................ | 6 | St. Martin ................. | 209 |
| St. Clement's ............. | 19 | St. Mark's ................. | 1 |
| Deptford .................. | 2 | Pimlico .................... | 10 |
| St. Dunstan's ............. | 1 | St. Pancras ................ | 245 |
| St. George's............... | 276 | Paddington ................. | 43 |
| St. George's, Bloomsbury .... | 78 | Pentonville ................ | 4 |
| Hyde ...................... | 1 | St. Swithin ................ | 1 |
| Islington ................. | 3 | Vauxhall ................... | 6 |
| St. John's ................ | 1957 | Wallworth .................. | 6 |
| | | Total ........ | 8475 |

| | | |
|---|---|---|
| Admitted under 3 years of age, 3115. | Admitted under 6 years of age, 2982. | Admitted under 12 years of age, 2378. |

